# Meeting of the American Medical Association

**Published:** 1860-03

**Authors:** Stephen G. Hubbard

**Affiliations:** Secretary, New Haven, Ct.


THE AMERICAN MEDICAL ASSOCIATION will hold its Thirteenth Annual Meeting at New Haven, on the *first Tuesday of June*, 1860.

The Secretaries of local Societies, Colleges, and Hospitals, are requested to forward to the undersigned the names of delegates, as soon as they are appointed. STEPHEN G. HUBBARD, M.D., *Secretary*, New Haven, Ct.

— The annual meeting of the New York State Medical Society, a complete report of the proceedings of which will be found in our pages, was one of unusual interest. The attendance, it is said, was larger than at any previous meeting of the Society. The inaugural address given by Dr. Barker, which we are obliged to omit on account of want of space, was eminently sound, and contained many practical suggestions. The closing remarks of the address, in which he expressed a hope "that the meetings of the session might be distinguished for the number and value of its papers, for the ability and interest of its discussions, for the wisdom and discrimination of its legislative acts, and for the harmony of feeling and personal friendships which it engenders," was fully realized, and doubtless much of it was due to the exertions of the presiding officer. The forthcoming volume of Transactions will be a very valuable one.

— Two new medical journals have reached us during the last month. One from California, entitled *The San Francisco Medical Press*, and edited by Dr. E. S. Cooper, Professor of Anatomy and Surgery in the University of the Pacific. It is to appear every other month, and contains sixty-four pages, at $2.00 a year. Dr. Cooper is well known, from his frequent contributions to various medical journals this side of the Rocky Mountains, and a journal under his direction cannot fail to give much and varied information.

The other new journal is edited by an old contributor to our own pages, and one who has done much service to the profession by his pen, as former editor to a valued Western medical journal. We are glad to welcome back Dr. Thos. W. Colescott to the pleasures of the editorial chair, and sincerely hope its trials may be lightened to him. The journal which he announces is called the *Louisville Medical Journal*, and is to be published monthly, containing sixty-four pages, at $3.00 a year. The first number gives promise of a valuable series.

— Dr. R. B. Todd, the eminent physiologist and physician, died the 30th of January last, in the 51st year of his life, from hæmorrhage of the stomach.

Dr. Todd was the son of a distinguished surgeon and professor in Dublin. He graduated at Trinity College of that city, went at an early age to London, and joined the College of Physicians. Soon after entering upon the duties of his profession in London he projected a work of great extent and reputation, the "Cyclopædia of Anatomy and Physiology," which was but recently completed. He also, with Mr. Bowman, commenced a work on the "Physiological Anatomy and Physiology of Man," which was also recently completed with the additional aid of Dr. Lionel S. Beale, the extensive and laborious practice into which Dr. Todd had gradually worked not affording sufficient time to devote to those minute investigations the subject demanded, and which had been the pleasure of the earlier and more quiet years of his professional life.

— The *Louisville Medical News* now appears once a month, instead of semi-monthly, and has changed its title accordingly. In every other respect it is the same as before.

